# MASMUT, la carte de visite de la coopération belge au développement en matière de droit à la santé et à la protection sociale

**DOI:** 10.48327/mtsimagazine.n1.2021.79

**Published:** 2021-04-02

**Authors:** A. Coheur, M. Hagiefstratiou, C. Horemans, L. Speecke

**Affiliations:** 1 Direction des Affaires Européennes et Internationales, Mutualité Solidaris; 2 Expertise internationale, Mutualités Libres; 3 Service Coopération Internationale, Mutualités Chrétiennes

**Keywords:** Mutuelles, Couverture santé universelle, Protection sociale, Afrique, Coopération, Mutual insurance, Universal health coverage, Social protection, Africa, Cooperation

## Abstract

L'objectif visé par les partenaires de MASMUT (Mon Assurance Santé Mutuelle) est d'assurer le droit effectif à la protection sociale et à la santé et de soutenir le rôle clé des mutuelles de santé pour y parvenir. Depuis 2014 et avec le soutien du gouvernement belge, ils appuient le développement du mouvement mutualiste dans plusieurs pays d'Afrique centrale et de l'Ouest, incluant des dynamiques régionales. Aujourd'hui, la crise de la Covid-19 nous démontre l'importance vitale, l'urgence de la protection sociale et de la santé confirmant encore davantage la pertinence des mutuelles et de leur extension. Des années cruciales s'annoncent pour les mutuelles africaines, et MASMUT continuera à accompagner leur évolution en synergie avec ses partenaires internationaux, l'AIM et le PASS.

## Abstract (en)

The objective aimed at by the MASMUT (Mon Assurance Santé Mutuelle) partners is to ensure the effective right to social protection and health and to support the key role of mutual health organizations in this achievement. Since 2014 and with the support of the Belgian government, they have been supporting the development of the mutualist movement in several Central and West African countries, including regional dynamics. Today, the Covid-19 crisis demonstrates the importance of social protection and health further confirming the relevance of mutual insurances and their extension. Crucial years lie ahead for African mutual insurances, and MASMUT will continue to accompany their evolution in synergy with its international partners, AIM and PASS.

## Introduction

MASMUT possède une longue histoire. Engagées volontairement dans un effort de structuration, de coordination et de complémentarité, les mutualités, les organisations non-gouvernementales (ONG), les institutions de recherche et la Coopération belge au développement, travaillant, en Belgique, dans le champ de la protection sociale, ont mis en place, il y a une dizaine d'années, un cadre de concertation, de mutualisation de l'expertise et d'actions communes, la « Plateforme MASMUT », afin de renforcer les systèmes de micro-assurance santé et de mutuelles de santé dans les pays africains, et de faire du droit à la santé une réalité.

Considérant la privatisation et la marchandisation de la santé et des soins comme une menace permanente pour les populations, les membres de MASMUT prônent l'intégration des mutuelles de santé dans la couverture sanitaire universelle en tant que mécanisme social et solidaire d'amélioration de la santé et d'accès aux soins de santé de qualité et abordables, ainsi que comme mouvement social. Et, dans cette perspective, l'approche de MASMUT se construit autour des objectifs de reconnaissance, de responsabilisation des acteurs de terrain et de renforcement de leurs compétences. Elle vise, *in fine,* l'autonomie des partenaires locaux ainsi que la capacité à porter directement les projets.

## Le début de MASMUT

Aux côtés d'autres acteurs de la coopération non gouvernementale, notamment des ONG, les mutualités sont devenues de véritables partenaires du développement. Forte de ce constat, la Belgique a, dans le cadre de la modernisation de sa coopération au développement, visé à intégrer, explicitement ou implicitement, l'ensemble des acteurs de la coopération belge, qu'ils soient gouvernementaux ou non-gouvernementaux. À ce titre, les mutualités, leur rôle et leur collaboration avec les ONG ont été reconnus en 2013 par la Loi relative à la Coopération au Développement.

Appréhendant cette reconnaissance comme une opportunité de se mobiliser en faveur du renforcement des dynamiques mutualistes sur le continent africain, les trois mutualités

- les Mutualités Chrétiennes, les Mutualités Socialistes - Solidaris, les Mutualités Libres
- et les ONG avec lesquelles elles sont engagées depuis de longues années - WSM, SolSoc, Louvain Coopération - ont décidé de déposer un programme commun et unique, le « Programme MASMUT 2014 - 2016 - Les mutuelles, acteurs et partenaires de la couverture santé universelle », au financement de la Coopération belge, couronné de succès.

Cette collaboration a continué avec la Synergie MASMUT 2017 - 2021, reposant sur une coalition des mêmes trois ONG et trois mutualités belges, en partenariat avec les acteurs mutualistes de huit pays africains: le Bénin, le Burkina Faso, la Guinée, le Mali, le Sénégal, le Togo, la République Démocratique du Congo et le Burundi.

## Une vision à long terme

La mobilisation des membres de la Synergie MASMUT s'inscrit dans une vision à long terme, celle de mouvements mutualistes forts, dynamiques et compétents qui, en Afrique de l'Ouest et en Afrique Centrale, contribuent de façon décisive à la mise en place et à la gestion d'une couverture sanitaire universelle. Est ainsi promue une stratégie intégrée de réalisation des socles nationaux de protection sociale et de la couverture sanitaire universelle, deux concepts étroitement imbriqués pour lesquels les mutualités, et leur gouvernance, représentent une réelle valeur ajoutée.

Dans les huit pays où MASMUT soutient le mouvement mutualiste, les actions menées en vue d'atteindre les objectifs visent:

- les plateformes mutualistes nationales et régionales en création ou existantes;
- les organisations régionales en Afrique (Union Économique et Monétaire Ouest-Africaine – UEMOA,…) impliquées dans la définition, le déploiement et l’évaluation de cadre légaux pour les mutuelles de santé;
- les administrations publiques en charge de la protection sociale en santé, que ce soit dans les pays africains, aux niveaux belge ou international;
- les pouvoirs publics dans les pays africains, en Belgique et à l'international;
- les populations exclues ou marginalisées des systèmes de protection sociale et d'assurance santé, par le biais de leur adhésion à une mutuelle de santé;
- plus largement, l’élaboration et/ou la consolidation de systèmes nationaux d'assurance maladie.

Eu égard à ce qui précède, la Synergie poursuit plusieurs objectifs comme la durabilité sociale. Les organisations partenaires (plateformes, mutuelles, structures d'appui), engagées dans la réalisation des différents objectifs spécifiques, sont des mouvements sociaux, démocratiques, autonomes et représentatifs. De ce fait, la légitimité de leurs actions par rapport aux publics cibles (principalement, la population) est assurée. De plus, du point de vue de la durabilité dite « socioculturelle et institutionnelle », elle repose sur le fait qu'il s'agit de structures endogènes, faisant partie intégrante de la société, et non de dispositifs exogènes qui auraient été créés de toutes pièces uniquement pour un programme. La durabilité politique est le deuxième objectif. Axée sur la thématique « Mutuelles de santé et couverture sanitaire universelle », la Synergie vise le renforcement de plateformes nationales d'acteurs mutualistes les plus larges possibles pour permettre une action concertée et, ainsi, pour peser en force sur les politiques de protection sociale et d'accès à la santé. Finalement, il y a la durabilité financière, car les plateformes des acteurs mutualistes doivent, à terme, être autonomes financièrement et leur fonctionnement, pris en charge par leurs membres et des ressources propres.

La vision de MASMUT s'inscrit aussi parfaitement dans les objectifs de l'Agenda 2030 pour le développement durable lancé par les Nations Unies en 2015. Dans cet Agenda, le droit à la santé est repris de manière transversale et la protection sociale est explicitement mentionnée dans l'Objectif 1 sur la lutte contre la pauvreté, plus spécifiquement dans la cible 1.3: « *Mettre en place des systèmes et mesures de protection sociale pour tous, adaptés au contexte national, y compris des socles de protection sociale, et faire en sorte que, d'ici à 2030, une part importante des pauvres et des personnes vulnérables en bénéficient* ». En outre, l'Objectif de développement durable (ODD) 3 sur la santé et le bien-être, l'ODD 5 sur le genre et l'ODD 10 sur les inégalités mentionnent également la protection sociale. Un lien peut également être fait avec d'autres objectifs inscrits dans cet Agenda, comme l'ODD 8 sur le travail décent. Le développement du mouvement mutualiste contribue à la réalisation de l'Agenda et de l'ensemble des objectifs.

L’ « Agenda du Travail Décent » et son pilier 3, « Protection sociale », sont des thèmes prioritaires de l'action de la Belgique en matière de coopération au développement et avec les pays partenaires. MASMUT est directement impliquée dans la Plateforme belge de Coordination Travail Décent (PCTD), son groupe de travail consacré à la protection sociale et son groupe de travail ciblant le « Continental - Afrique ».

## Résultats

Il reste encore d'importants défis à relever, mais MASMUT a pu contribuer ces dernières années au renforcement du mouvement mutualiste en Afrique Centrale et de l'Ouest. Ses résultats portent sur la structuration du mouvement, le renforcement des capacités techniques des mutuelles, l'analyse et capitalisation des acquis et le plaidoyer politique.

La **structuration** du mouvement mutualiste est un premier chantier important de la Synergie MASMUT. Dans les 8 pays où MASMUT soutient le mouvement mutualiste, il existe aujourd'hui une plateforme nationale représentative des mutuelles et de la vision solidaire et inclusive qu'elles portent. Toutes les plateformes sont reconnues légalement, disposent d'un siège et sont dotées d'un secrétariat permanent. Elles sont également identifiées comme un interlocuteur de référence du gouvernement de leur pays et du dialogue structuré organisé avec la société civile:

- au Bénin: Conseil National béninois des Structures d'Appui aux Mutuelles Sociales (CONSAMUS);
- au Burkina Faso: Concertation des Acteurs de la Mutualité Sociale au Burkina Faso (CAMUS/BF);
- au Burundi: Plateforme des Acteurs des Mutuelles de santé au Burundi (PAMUSAB);
- en Guinée: Plateforme des Promoteurs de la Protection Sociale en Guinée (PPSOGUI);
- au Mali: Union Technique de la Mutualité Malienne (UTM);
- au Sénégal: Union Nationale des Mutuelles de Santé Communautaire du Sénégal (UNAMUSC);
- au Togo: Cadre National de Concertation de la Mutualité (CNCMUT);
- en RDC: Plateforme des Organisations des Mutuelles de santé du Congo (POMUCO);
- pour l'Afrique de l'Ouest et, plus récemment, pour l'Afrique Centrale, des coordinations zonales sont déployées.

La consolidation de la structuration est toujours en cours, en vue d'atteindre un double objectif important, la création d'Unions et l'articulation entre les niveaux de gouvernance, qui renforce la collaboration entre les mutuelles.

Le renforcement des capacités techniques des mutuelles de santé est un deuxième projet sur lequel MASMUT a beaucoup travaillé ces dernières années. Les mutualités veulent jouer un rôle dans la couverture sanitaire universelle sur laquelle de nombreux pays sont mobilisés. Cela signifie qu'elles doivent pouvoir se présenter comme des partenaires fiables, à la gestion robuste et bien organisés. La professionnalisation des mutuelles est donc cruciale, et des étapes importantes ont été franchies dans cette direction. Des formations ont été organisées pour les mutualistes dans les différents pays, couvrant des sujets tels que la gestion financière d'une mutualité, le rôle des médecins-conseils, le marketing social et l'importance du plaidoyer politique. Diverses formations ont été organisées en collaboration avec l'Université de Versailles Saint-Quentin-en-Yvelines. Des outils de gestion et de communication ont également été développés afin de renforcer et d'améliorer l'efficacité des services des mutualités, et afin que celles-ci disposent rapidement de données. L'implémentation du logiciel « SIGMA » - Système Intégré d'Information et de Gestion des mutuelles de santé - est à l'ordre du jour dans plusieurs pays.

Pour le développement futur des mutuelles africaines, il est important de capitaliser les expériences et de partager les bonnes pratiques (« best practices »). En outre, il est essentiel de mieux comprendre le fonctionnement des mutuelles et d'analyser comment elles peuvent évoluer afin de jouer un rôle dans la couverture universelle. Plusieurs études ont été réalisées par les membres de la Synergie MASMUT, telles que « *L'avenir des mutuelles de santé au Bénin et Togo - Comment optimiser l'utilisation des données des mutuelles? Comment convaincre la population de devenir membre et de le rester?^1^»* et « *Étude des mutuelles de santé en RDC dans le cadre de la couverture sanitaire universelle^2^* ».

Enfin, de grands efforts ont également été déployés dans les différents pays et de manière régionale en termes de plaidoyer politique. Au niveau national, ce sont les plateformes nationales qui interpellent le gouvernement de leur pays afin de mettre en œuvre la réglementation de l'UEMOA concernant la mutualité sociale, et d'intégrer les mutuelles de santé dans l'architecture de la couverture santé de leur pays. Dans des pays tels que le Sénégal et le Mali, cet objectif a d'ailleurs été réalisé dans une large mesure. L'invitation d'un représentant de la Synergie MASMUT à participer aux travaux du Comité consultatif de la mutualité sociale de la Commission de l'UEMOA a également été une avancée à saluer.

Les conférences internationales organisées par la Synergie MASMUT sous l’égide de l'AIM (Association Internationale de la Mutualité) et avec le PASS (Programme d'Appui aux Stratégies Sociales), ont bien sûr constitué des jalons remarquables. La première fut tenue à Abidjan en 2016, puis à Lomé en 2019. D'importantes délégations du mouvement mutualiste d'Europe et d'Afrique ont participé à ces événements, qui ont représenté des signaux très forts pour le monde politique, avec la demande d’œuvrer à la reconnaissance des mutuelles et l'invitation à se mobiliser tous ensemble pour une meilleure protection sociale des populations. Par ailleurs, lors de la Conférence de Lomé sur le thème « *Le Pari de la Mutualité pour le XXI^e^ siècle* », le document « *Plateforme de Lomé^3^* » a été approuvé à l'unanimité par les participants. Ce cahier de revendications politiques est aujourd'hui la feuille de route stratégique du mouvement mutualiste des pays africains. Les partenaires de MASMUT en ont fait une priorité dans leur plan d'action: restitutions nationales, suivi-évaluation des mesures adoptées au regard des axes stratégiques… Bref, passer des mots aux actes.

## Mutuelles et COVID-19

L'Afrique a aussi été touchée par le Coronavirus. Fin octobre 2020, l'Organisation mondiale de la santé (OMS) a fait état de 1,6 million d'infections et de 40 000 décès. Depuis le début de la crise, les partenaires de la Synergie MASMUT en Europe et en Afrique ont uni leurs forces pour endiguer la propagation du virus. Les plateformes mutualistes et leurs membres ont adapté leur plan d'action et mis en œuvre des dispositifs opérationnels sur le terrain. Par exemple, concernant l'information et la sensibilisation, les mutuelles de santé ont contribué à diffuser les messages sanitaires des autorités nationales. Il s'agissait notamment de rappeler les mesures préventives par des communications pédagogiques lors d’émissions de radio et lors de campagnes de sensibilisation, ainsi que de construire des installations pour le lavage des mains. De plus, les partenaires de la Synergie MASMUT ont également organisé des échanges sur les expériences des mutualistes concernant l'impact de la Covid-19. Chaque membre de la Synergie a également pris des initiatives et les a partagées: webinaire^4^ sur l'impact de la crise de la Covid-19 sur le mouvement mutualiste dans différents pays africains…

Au final, la pandémie ne pourrait-elle pas constituer un élément déclencheur et amener une redistribution des priorités? Qui sait, cette situation va-t-elle peut-être convaincre les gouvernements et les bailleurs internationaux d'investir davantage dans la protection sociale et dans la santé et ce, de manière coordonnée? Une fois que cette pandémie sera derrière nous, et pour une relance durable et inclusive, les partenaires MASMUT demandent d'accorder effectivement une place renforcée à la protection sociale et au droit à la santé dans la coopération internationale belge. La Belgique devrait également défendre plus résolument ce thème dans les institutions internationales. Le soutien à un Fonds Mondial pour la Protection Sociale est, à titre illustratif, une des pistes portées par MASMUT et ses réseaux.

Mais quel sera l'impact de la crise du coronavirus sur les mutuelles de santé africaines? Quelle sera l'ampleur des dégâts? La crise affecte aussi les revenus de la population. Pourra-t-elle encore payer ses cotisations aux mutuelles de santé? Avant la crise, de nombreuses mutuelles de santé africaines étaient déjà dans une situation financière fragile.

## Conclusion

Des années cruciales s'annoncent donc pour le mouvement mutualiste en Afrique. La poursuite de la réalisation d'une couverture sanitaire universelle et de soins de santé de qualité sera sans aucun doute une priorité pour de nombreux pays africains. Les partenaires MASMUT restent convaincus que les mutuelles peuvent jouer un rôle particulièrement précieux dans cette évolution. Toutefois, elles ne pourront le faire que s'il existe une volonté politique de soutenir le mouvement mutualiste en lui confiant la délégation de gestion, en l'assortissant d'un soutien financier pérenne et en instaurant un système d'affiliation obligatoire.

C'est pourquoi les partenaires MASMUT continueront lors de la prochaine Synergie MASMUT, 2022 - 2026, à soutenir leurs partenaires africains pour réaliser ces ambitions et à collaborer, avec l'AIM, le PASS… « *Si vous voulez aller vite, allez-y seul. Si vous voulez aller loin, allez-y ensemble* » et, ensemble, c'est indispensable pour ne laisser personne de côté (« Leaving no one behind »).

## Conflits d'intérêts

Les auteurs ne déclarent aucun conflit d'intérêts.

